# Reference-free, high-resolution measurement method of timing jitter spectra of optical frequency combs

**DOI:** 10.1038/srep40917

**Published:** 2017-01-19

**Authors:** Dohyeon Kwon, Chan-Gi Jeon, Junho Shin, Myoung-Sun Heo, Sang Eon Park, Youjian Song, Jungwon Kim

**Affiliations:** 1 School of Mechanical and Aerospace Engineering, Korea Advanced Institute of Science and Technology (KAIST), Daejeon 34141, Korea; 2 Center for Time and Frequency, Korea Research Institute of Standards and Science (KRISS), Daejeon 34113, Korea; 3 Science of Measurements Program, University of Science and Technology (UST), Daejeon 34114, Korea; 4 School of Precision Instruments and Opto-electronics Engineering, Tianjin University, Tianjin 300072, China

## Abstract

Timing jitter is one of the most important properties of femtosecond mode-locked lasers and optical frequency combs. Accurate measurement of timing jitter power spectral density (PSD) is a critical prerequisite for optimizing overall noise performance and further advancing comb applications both in the time and frequency domains. Commonly used jitter measurement methods require a reference mode-locked laser with timing jitter similar to or lower than that of the laser-under-test, which is a demanding requirement for many laser laboratories, and/or have limited measurement resolution. Here we show a high-resolution and reference-source-free measurement method of timing jitter spectra of optical frequency combs using an optical fibre delay line and optical carrier interference. The demonstrated method works well for both mode-locked oscillators and supercontinua, with 2 × 10^−9^ fs^2^/Hz (equivalent to −174 dBc/Hz at 10-GHz carrier frequency) measurement noise floor. The demonstrated method can serve as a simple and powerful characterization tool for timing jitter PSDs of various comb sources including mode-locked oscillators, supercontinua and recently emerging Kerr-frequency combs; the jitter measurement results enabled by our method will provide new insights for understanding and optimizing timing noise in such comb sources.

Optical frequency combs have evolved to be a powerful tool for various high-precision applications ranging from optical atomic clocks^1^ through frequency-domain spectroscopy^2^ to astro-combs^3^. Timing jitter (i.e., phase noise in pulse repetition-rate)^4^ is one of the most important properties of femtosecond mode-locked lasers and optical frequency combs. First, there are applications where the pulse timing jitter directly impacts the achievable performance, such as low-phase noise microwave generation^5^,^6^,^7^, timing synchronization for X-ray free-electron lasers^8^,^9^, pulse time-of-flight-based ranging^10^, photonic analogue-to-digital converters^11^, photonics-based radars^12^, and clock distribution networks^13^, to name a few. Timing jitter, in fact, also significantly contributes to the optical linewidth of optical frequency comb lines and phase noise of carrier-envelop-offset frequency (*f*_ceo_) in the frequency domain^14^,^15^,^16^. Thus, accurate measurement of timing jitter power spectral density (PSD) is an important prerequisite for optimizing the jitter performance and further advancing frequency comb applications both in the time and frequency domains. However, complete characterization of timing jitter PSD in frequency combs (including mode-locked laser oscillators and their supercontinua) is often challenging and complicated. This difficulty frequently limited the accurate assessment of timing jitter performance of mode-locked lasers and frequency comb sources in laser laboratories.

Conventional timing jitter measurement methods are based on microwave phase detector method^17^,^18^. Optical pulse train generated from the laser-under-test (LUT) is converted to a microwave signal using a high-speed photodiode and a band-pass filter, and its phase is compared with the reference microwave signals for phase noise (i.e., equivalent pulse timing jitter) measurement. This microwave phase detector method is very convenient, and even commercial signal source analysers can be used. However, measurement resolution is limited by both the reference oscillator phase noise and additional phase noise added in the photo-detection process. Typical shot noise-limited measurement noise floor in photo-detection is ~−140 dBc/Hz level at 10-GHz carrier when detecting a ~100-MHz repetition-rate laser^19^. In addition, amplitude-to-phase conversion in the photodiode also adds excess timing jitter^20^,^21^,^22^. This noise floor is too high for timing jitter characterization of mode-locked lasers, because repetition-rate phase noise of free-running mode-locked lasers is often well below −140 dBc/Hz from 10 kHz Fourier frequency (when scaled to 10-GHz carrier)^23^,^24^,^25^. In order to improve the detection resolution, optical cross-correlation method^19^,^23^,^24^,^25^ can be used; this technique makes use of direct pulse-to-pulse timing comparison between two optical pulse trains using nonlinear optic processes (such as second-harmonic generation). When shorter pulse width and higher power are used, measurement noise floor of the optical cross-correlation can be improved. For example, when 50 mW average power and 60 fs pulse width were used, timing jitter could be measured with 3 × 10^−12 ^fs^2^/Hz (−202 dBc/Hz at 10-GHz carrier) background noise floor^23^. More recently developed optical heterodyne method, which measures timing jitter using optical spectrum interference between two identical mode-locked lasers, showed −212 dBc/Hz background noise floor at 10-GHz carrier^26^. Although the optical cross-correlation and optical heterodyne methods show the best timing jitter measurement resolution of mode-locked lasers, these methods require two identical mode-locked lasers or a reference mode-locked laser with matched repetition-rate and lower timing jitter, which is often a difficult requirement to meet. Thus, a high-resolution and reference-free timing jitter measurement method is highly desirable for simple yet accurate characterization of various optical frequency comb sources.

In microwave engineering community, delay-line-based methods have enabled reference-source-free measurements of phase noise in microwave and radio-frequency (RF) oscillators^27^. This method can be directly applied to measure the timing jitter and phase noise of mode-locked lasers by using optical fibre link as the delay line. However, the measurement noise floors have been limited to ~−140 dBc/Hz at 10-GHz carrier by limited signal power and thermal noise in photodiodes, RF amplifiers, and frequency mixers^28^. Even when optical cross-correlation was used as a sensitive timing detector, the measurement resolution was hampered by the dispersion and nonlinearities in long fibre links, which limited the achievable pulsewidth and pulse energy^19^,^29^.

In this paper, we demonstrate a high-resolution and reference-source-free measurement method of timing jitter spectra in optical frequency combs using an optical fibre delay line and optical carrier interference. The basic principle is based on the optical carrier frequency interference using a fibre delay line, which has been used for the measurement and stabilization of continuous-wave (CW) laser frequency noise^30^,^31^,^32^,^33^, to measure the timing jitter of optical frequency combs. Note that, as this method uses optical carrier interference, it does not require clean ultrashort pulses in the time domain like the optical cross-correlation method. As a result, the demonstrated method can conveniently measure timing jitter of both mode-locked laser oscillators and spectrum-broadened supercontinua with 2 × 10^−9 ^fs^2^/Hz (−174 dBc/Hz at 10-GHz carrier frequency) measurement noise floor. Therefore, the demonstrated method can serve as a simple and powerful characterization tool for timing jitter PSDs of various supercontinuum sources^34^,^35^ and recently emerging Kerr-frequency comb sources^36^,^37^ as well; the measurement results will provide new insights for understanding noise mechanisms and further optimizing noise performances in such comb sources.

## Results

### Design and implementation of the timing jitter PSD measurement setup

**Figure 1:**
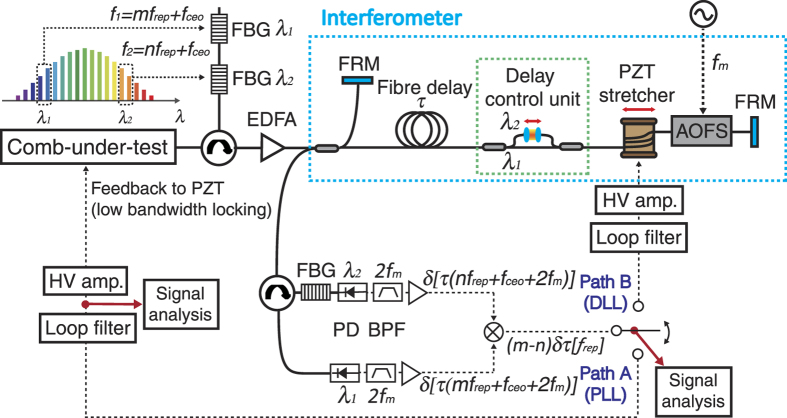
Schematic of the fibre delay-line-based timing jitter measurement method. PZT, piezo-electric transducer; FBG, fibre Bragg grating; EDFA, Erbium-doped fibre amplifier; FRM, Faraday rotating mirror; AOFS, acousto-optic frequency shifter; PD, photodetector; BPF, band-pass filter; PLL, phase-locked loop; DLL, delay-locked loop; HV amp, high voltage amplifier.

Timing jitter measurement is based on optical carrier interference by an all-fibre Michelson interferometer with a long optical fibre delay. Figure 1 shows the experiment setup. Using the optical carrier interference between a reference arm and a long delay arm, the absolute frequency noise of comb modes (*nf*_rep_ + *f*_ceo_) is detected. To extract only the repetition-rate noise, the carrier-envelope-offset frequency (*f*_ceo_) noise is eliminated by common-mode *f*_ceo_ rejection using two spectral regions (λ_1_ and λ_2_ in Fig. 1) of the laser^38^,^39^,^40^. We convey all the frequency noise signals into RF frequency *2 f*_*m*_ using an acousto-optic frequency shifter (AOFS). At the Michelson interferometer output, each wavelength component (λ_1_ and λ_2_) is split and detected by photodetectors. Each photodetected power contains the frequency noise of the corresponding frequency modes (i.e., (*mf*_*rep*_ + *f*_*ceo*_ + *2 f*_*m*_) for λ_1_ and (*nf*_*rep*_ + *f*_*ceo*_ + *2 f*_*m*_) for λ_2_) at *2 f*_*m*_ carrier frequency (i.e., twice the AOFS driving frequency by round-trip). After the common frequency components are rejected by a frequency mixer, the repetition-rate frequency noise, *δ(m* − *n)f*_*rep*_, is detected. Once the frequency noise is obtained, it can be easily converted to the equivalent phase noise and timing jitter. Note that a similar fibre interferometer-based method was recently used for stabilization of repetition-rate phase noise^38^. In this work, we focus on the use of the optical fibre delay for analysing the timing jitter PSDs in free-running mode-locked laser oscillators and their supercontinua. More detailed information on the experiment setup can be found in the Methods Section.

We modified the previous fibre Michelson interferometer setup to make it more suitable for timing jitter measurement of various frequency comb sources. First, in order to make the measurement setup both wavelength- and repetition-rate-independent, we replaced the dispersion compensating fibre (DCF) in the fibre link (used in ref. 38) with a delay control unit consisting of a pair of wavelength division multiplexing (WDM) couplers and a variable delay line (see “delay control unit” in Fig. 1). This enables easy adjustment of relative group delay between λ_1_ and λ_2_ components, which enables the highest possible signal-to-noise ratio (SNR) at the interferometer output. As DCF is not necessary for building the long fibre link, the jitter measurement setup can be built for wavelength ranges other than 1550-nm (for example, Yb-fibre combs at 1-μm or Ti:sapphire combs at 800-nm). In addition, the high-SNR interference condition can be easily found for any comb repetition-rates by simply tuning the relative delay between the two wavelengths.

**Figure 2:**
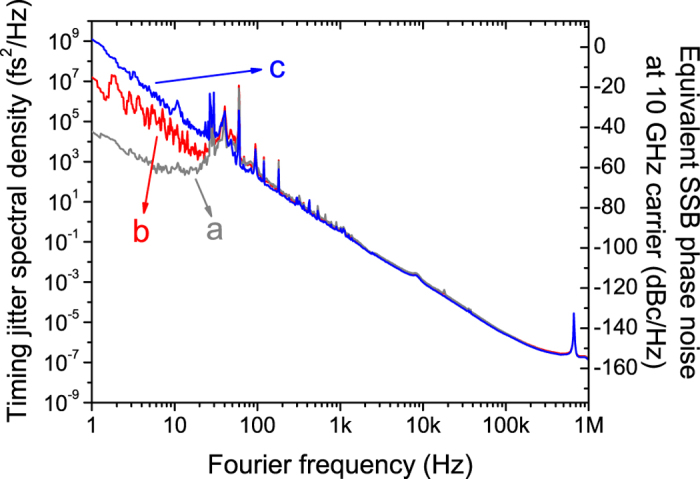
Comparison of timing jitter PSD measurement results between PLL and DLL approaches. (**a**) Timing jitter measured at the mixer output of the PLL. (**b**) Timing jitter measured at the mixer output of the DLL. (**c**) Timing jitter by combining the measurement results at the mixer output and the laser PZT input of the PLL (using the method in ref. 41).

Second, we showed that both phase-locked loop (PLL) and delay-locked loop (DLL) approaches can measure the timing jitter of optical frequency combs. As shown in Fig. 1, the comb repetition-rate frequency noise information can be fed back to either the comb-under-test (via path A in Fig. 1) or the fibre delay line (via path B in Fig. 1), which corresponds to the PLL and DLL configuration, respectively. The advantage of PLL approach is that it can measure the timing jitter PSD inside the locking bandwidth as well by monitoring the PSD of the voltage signal that drives the repetition-rate-tuning actuator in the comb source, which carries the frequency noise information of the free-running comb^41^. One important requirement of using PLL approach is that the comb-under-test should have a repetition-rate-tuning actuator such as piezoelectric transducer (PZT)-mounted optics. In case of the mode-locked oscillator or comb source without such actuators, the DLL approach can be employed. In this case, the error signal is fed back to the PZT stretcher in the fibre link, and the timing delay provided by the long fibre link is locked to the free-running comb. As a result, the timing jitter PSD of the free-running comb can be measured outside the locking bandwidth. Figure 2 shows the timing jitter PSD measurement results of the free-running mode-locked Er-fibre oscillator in a commercial comb system (FC1500 from MenloSystem GmbH manufactured in 2010) using a 140-m-long fibre link. Curves **a** and **b** show the timing jitter PSDs measured at the mixer output using the PLL and DLL approaches, respectively, when the locking bandwidth is set to ~70 Hz. Both methods show consistent and valid jitter measurement results above the locking bandwidth. Curve **c** shows the timing jitter PSD for the entire Fourier frequency range by combining the measurement results at the mixer output and the laser PZT input when the PLL approach is used (by using the approach shown in ref. 41).

**Figure 3:**
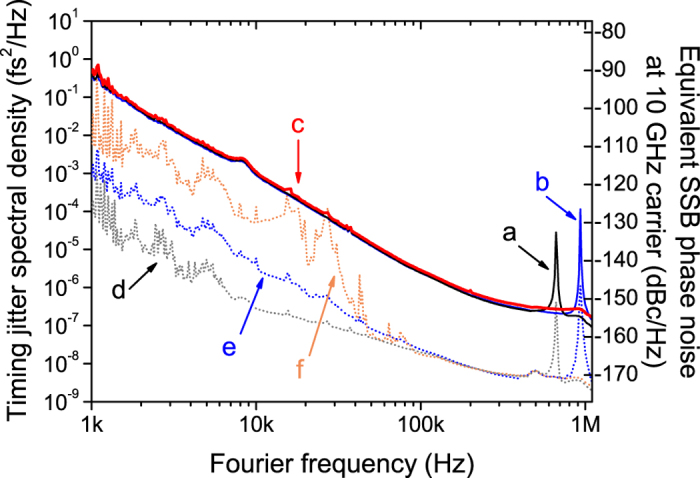
Comparison of timing jitter PSD measurement results for different fibre delay lengths. (**a**) 140-m-long fibre link result. (**b**) 100-m-long fibre link result. (**c**) 45-m-long fibre link result. (**d**) Background noise floor of 140-m-long fibre link. (**e**) Background noise floor of 100-m-long fibre link. (**f**) Background noise floor of 45-m-long fibre link.

Third, we showed that fibre delay length can be optimized for comb sources with different noise characteristics. When the fibre delay time is τ, the jitter measurement sensitivity scales with τ, which enables lower measurement background noise for longer delay. However, the frequency noise detection sensitivity is nullified for Fourier frequencies at 1/τ and its harmonics^28^,^38^. As a result, multiple spikes at 1/τ and its harmonics appear in the measured timing jitter spectrum, which are not the real comb noise but simply a measurement artefact (see Methods section and Supplementary Information of ref. 38 for more discussion). Thus, for a given comb-under-test, a trade-off between the measurement sensitivity (which scales with τ) and the null-sensitivity frequency position (which scales with 1/τ) is necessary. It is generally desirable to reduce the delay length down to the point where the background noise level is slightly lower than the jitter PSD of the comb-under-test, which results in the shift of the sensitivity null-point (1/τ) to higher Fourier frequency. The timing jitter PSD measurement results in Fig. 3 demonstrate the impact of different fibre delay lengths. We measured the timing jitter PSD of the aforementioned Er-fibre oscillator (in FC1500 of MenloSystems GmbH) using 140-m (curve **a**), 100-m (curve **b**), and 45-m (curve **c**) fibre delay lines. Note that curves **d**, **e** and **f** correspond to the measurement background noise for 140-m, 100-m, and 45-m fibre delay cases, respectively. As expected, the background noise increases for shorter fibre delay, and 45-m-long fibre is the shortest fibre length that can faithfully measure the jitter PSD for the comb-under-test. All three measurements show same level of jitter PSD measurement results except the measurement artefact spikes at the first null-point (1/τ). As the sensitivity null-point shifts from 680 kHz (140-m fibre) to 1 MHz (100-m fibre) and 2 MHz (45-m fibre), the measurement with 45-m-long fibre delay (curve **c**) enables spike-free measurement up to 1-MHz Fourier frequency.

### Timing jitter PSD measurement of low-jitter mode-locked oscillators

**Figure 4:**
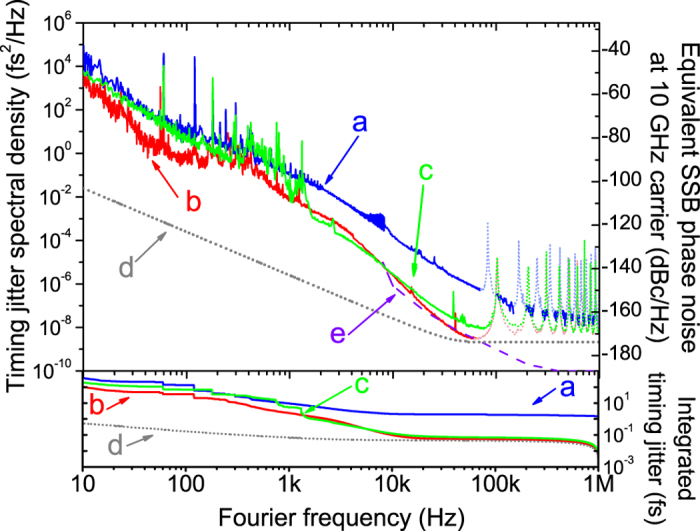
Timing jitter PSD and integrated rms timing jitter measurement results of sub-femtosecond-jitter free-running mode-locked oscillators. (**a**) Homebuilt Er-fibre mode-locked oscillator. (**b**) Commercial Er-laser oscillator (Origami-15 from Onefive GmbH). (**c**) Commercial Er-fibre oscillator (FC1500-250-ULN from MenloSystems GmbH). (**d**) Projected background noise floor originated from the RIN at 100 MHz. (**e**) Er-fibre oscillator jitter data shown in ref. 43 (measured by BOC method) for comparison.

Using the demonstrated measurement method, we characterized the timing jitter PSDs of low-jitter (e.g., sub-fs integrated jitter for >10 kHz Fourier frequency) mode-locked oscillators. We tested three different oscillators: First one is a home-built 78-MHz stretched-pulse Er-fibre laser oscillator^41^ (denoted as “Oscillator A”); the second one is a commercial 80-MHz Er-doped mode-locked laser oscillator (Origami-15 manufactured by Onefive GmbH, denoted as “Oscillator B”); the third one is a commercial 250-MHz nonlinear amplifying loop mirror (NALM)-based, polarization-maintaining (PM) Er-fibre laser oscillator (FC1500-250-ULN manufactured by MenloSystems GmbH, denoted as “Oscillator C”). Figure 4 shows the measured timing jitter PSDs and integrated rms timing jitter of the three mode-locked oscillators (curves **a**, **b** and **c** for Oscillator A, B and C, respectively) using a ~1-km-long fibre delay-based PLL method. Note that the sensitivity-null-point-induced measurement artefact spikes (at ~100 kHz and its harmonics) are represented by dotted curves in this plot. Curve **d** shows the projected measurement noise floor originated from the RIN at 100 MHz (2 *f*_*m*_). The measurement background noise floor is 2 × 10^−9 ^fs^2^/Hz (45 zs/√Hz), which corresponds to −174 dBc/Hz single-sideband phase noise floor at 10-GHz carrier frequency. The rms timing jitter integrated from 10 kHz to 1 MHz Fourier frequency is 861 as, 144 as and 175 as (when excluding the impact of artefact spikes) for Oscillator A, B and C, respectively, where the RIN-limited measurement background noise contributes 48-as rms jitter in the same integration range. Note that the characteristic steep slope of >40 dB/dec of Oscillator B in the 3 kHz–30 kHz Fourier frequency range was also previously observed in a measurement result of a similar oscillator using the BOC method^42^. Also note that, despite the 1/*f*^2^ increase in the measurement sensitivity (curve **d**), the rms background jitter is only 529 as when integrated from 10 Hz to 1 MHz. This background noise floor is low enough to measure the timing jitter PSD of even the lowest-jitter oscillators such as the Er-fibre oscillator demonstrated in ref. 43, the equivalent jitter PSD of which is shown by curve **e**.

### Timing jitter PSD measurement of supercontinuum

Many frequency comb applications require supercontinuum (SC) generation from the mode-locked oscillator output for the detection and stabilization of carrier-envelope-offset frequency (*f*_*ceo*_) via *f* − 2*f* interference. It is well known that SC generation process can suffer from significant fluctuations in carrier phase, timing and amplitude, mainly due to amplification of input laser noise and complex noise coupling during nonlinear propagation^34^,^35^,^44^,^45^. Low timing jitter in the SC generation is important because carrier-envelope phase noise itself is a linear combination of timing jitter and carrier phase noise^14^,^15^,^35^. There are also other applications where timing jitter of SC directly impacts the achievable performance, such as coherent optical pulse synthesis^46^,^47^ and coherent anti-Stokes Raman scattering (CARS) microscopy^48^. For these reasons, accurate measurement of SC timing jitter is desirable, however, high-resolution and accurate jitter measurement is often nontrivial. The phase detector method suffers from amplitude-to-phase conversion in photodetection; the optical cross-correlation method cannot be used for many cases (especially for octave-spanning SC generation for self-referencing) because the pulse shape of the SC output is often seriously deformed^34^. Thus, the fibre-delay-based method proposed in this paper can be an attractive alternative that can achieve high-resolution timing jitter measurement of SC sources.

**Figure 5:**
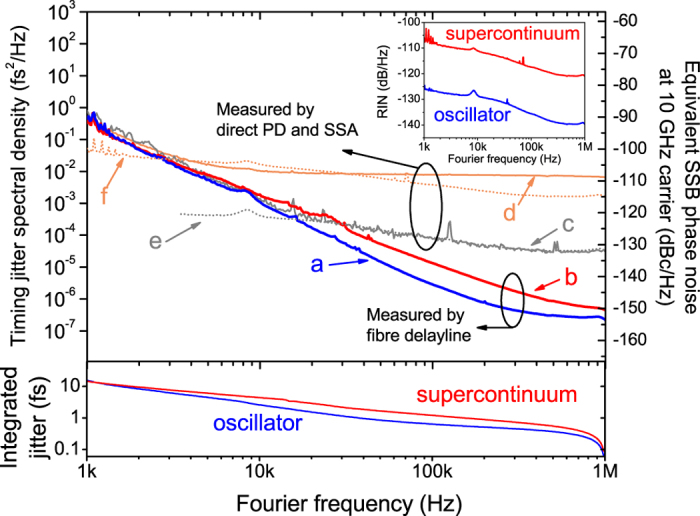
Timing jitter PSD and integrated rms timing jitter measurement results of the mode-locked laser oscillator and its supercontinuum output. (**a**) Timing jitter of the oscillator measured by the 45-m-long fibre link. (**b**) Timing jitter of supercontinuum output measured by the 45-m-long fibre link. (**c**) Timing jitter of the oscillator measured by direct photodetection and signal source analyser. (**d**) Timing jitter of the supercontinuum output measured by direct photodetection and signal source analyser. (**e**) Projected excess timing jitter by amplitude-to-phase conversion in photodetection for the oscillator. (**f**) Projected excess timing jitter by amplitude-to-phase conversion in photodetection for the supercontinuum. Inset shows the measured RIN spectra of the oscillator and supercontinuum outputs.

As a demonstration experiment, we measured the timing jitter PSDs of both the oscillator and SC outputs of a commercial frequency comb system (aforementioned FC1500 from MenloSystems GmbH). Figure 5 shows the measurement results. Curves **a** and **b** represent the timing jitter PSDs of the oscillator and the SC, respectively, measured by the proposed method using a 45-m-long fibre delay line. Note that, as already discussed in Fig. 3, the measured jitter spectra are not limited by the measurement noise floor and the 1/τ spike is at ~2 MHz for the 45-m-long fibre delay. As a result, the measured PSDs in Fig. 5 (below 1-MHz Fourier frequency) are free from any measurement artefacts. The timing jitter PSD of the SC is slightly higher than that of the oscillator by ~5 dB for >10-kHz Fourier frequency range. From this high-resolution measurement, the rms excess timing jitter in the SC generation is obtained as 580-as (1.62-fs) in the 100 kHz (10 kHz)–1 MHz integration range. Note that this excess jitter number agrees fairly well with the previous simulation prediction for SC generation^35^.

For comparison, we also used a direct photodetection (2-GHz InGaAs p-i-n photodiode) and a signal source analyser (Keysight Technologies, E5052B) to measure the timing jitter PSDs by the traditional phase detector method. Curves **c** and **d** represent the timing jitter PSDs of the oscillator and the SC, respectively, measured by the direct photodetection. Unlike the fibre delay-line-based measurement results, not only their PSD levels are much higher, but also the SC PSD is ~20 dB higher than the oscillator PSD. Both issues might be the result of large amplitude-to-phase conversion^20^,^21^,^22^ in the used photodiode. As shown in the inset, the relative intensity noise (RIN) of the SC is ~20 dB higher than that of the oscillator. When using the measured amplitude-to-phase conversion coefficient of the used photodiode (0.43 rad/mW at 1 GHz = 67 ps/mW), the projected RIN-converted jitter PSDs for the oscillator and the SC are curves **e** and **f**, respectively, which fit fairly well with the measured timing jitter PSDs with direct photodetection (curves **c** and **d**).

These measurement results show that our fibre-delay-based method enables high-resolution and accurate timing jitter PSD measurement of SC sources as well, which is not hampered by the large RIN in the SC process. To our knowledge, this is the first time to accurately characterised the excess timing jitter spectrum added in the SC process and quantified it with sub-100-as resolution over 1-MHz bandwidth.

## Discussion

In this report, we showed a reference-source-free and high-resolution timing jitter measurement method using an all-fibre Michelson interferometer. Timing jitter PSDs of both mode-locked oscillators and supercontinuum sources have been successfully measured with 2 × 10^−9 ^fs^2^/Hz noise floor (equivalent to −174 dBc/Hz phase noise floor at 10-GHz carrier), which is >25 dB lower than typical photodiode-based measurements^19^,^49^,^50^,^51^. Note that, although the noise floor is higher than the best optical cross-correlation method result, it is still comparable to typical performances of many optical cross-correlation measurements reported so far^19^,^52^,^53^,^54^.

Our demonstrated method can be particularly attractive for timing jitter characterization of SC sources and microresonator-based Kerr-comb sources. Precise measurement of excess timing jitter in SC is important for identifying different noise coupling mechanisms, such as amplitude-to-timing conversion^35^, Gordon-Haus jitter effect^55^, Raman soliton formation^56^ and soliton fission^57^, and further minimizing the excess jitter. As shown in Fig. 5 result, our method can provide a simple and high-resolution characterization of the timing jitter PSDs added in the SC generation.

There has been intense research in microresonator-based Kerr frequency combs in the last decade^36^,^37^. Due to the recent advancements in the generation of temporal soliton pulse trains^58^,^59^,^60^,^61^ at high repetition-rates in the tens – hundreds GHz range, low timing jitter Kerr-combs can be a useful source for telecommunication and photonic signal processing. Although the repetition-rate phase noise of Kerr-combs has been measured by standard photodiode-based methods, the measurement resolution has been often limited by the used microwave signal generators and photodetection^62^,^63^,^64^. As an alternative, our fibre-delay-based measurement method has a potential for rapid and accurate assessment of timing jitter performance for various operation conditions of Kerr-combs (such as pump detuning, pump power and group velocity dispersion). In particular, since many Kerr-combs are not equipped with direct repetition-rate tuning mechanism (such as PZTs), the DLL configuration will be useful for measuring the timing jitter of free-running combs without any feedback signal to the comb-under-test.

## Methods

### Design and implementation of the timing jitter measurement setup

The all-fibre Michelson interferometer-based timing jitter measurement setup is shown in Fig. 1. The setup works for a wide range of input optical power, and we could get high-resolution jitter measurement for input power as low as 400 μW. The input comb is filtered by fibre Bragg gratings (FBGs) with a full-width-half-maximum (FWHM) spectral width of 2 nm. The centre wavelengths of FBGs are selected for the best measurement SNR, depending on the optical spectrum of the comb-under-test: for example, 1540-nm and 1560-nm are used for home-built Er-fibre oscillator and FC1500-250-ULN oscillator, whereas 1550-nm and 1570-nm are used for Origami-15 oscillator in our experiment. Filtered comb is amplified to ~20 mW by an Erbium-doped fibre amplifier (EDFA), which is the power level that achieves the best SNR in our measurements. Note that, when the EDFA output power level is not optimized, undesired fibre-scattering-induced intensity noise limits the measurement resolution of the system.

Filtered and amplified comb is then applied to the Michelson interferometer with a long fibre delay. The length of the delay arm is adjusted from 45-m to 1-km depending on the intrinsic jitter level of the comb-under-test. The delay arm includes a fibre-coupled acousto-optic frequency shifter (Brimrose, AMF-50-5-1570-2FP) for synchronous detection at 2 *f*_*m*_ (100 MHz in this work). As the optical fibre has chromatic dispersion, the group delay for filtered comb spectra λ_1_ and λ_2_ is different: for example, for SMF-28e fibre with 17 ps/nm/km dispersion coefficient at 1550 nm, the difference of optical path length is ~340 ps when (λ_2_ − λ_1_) is 20 nm and delay length is 1 km. To enable concurrent interference of both λ_1_ and λ_2_ at the interferometer output, the group delays of λ_1_ and λ_2_ are matched by the delay control unit with two WDM couplers and a variable delay line (General Photonics, MDL-002-D-15-33-FC/APC-PP) inserted in one of the paths. In addition, a 40-m-long spool of PZT stretcher (Optiphase, PZ2-PM2-APC-E-155P) is inserted in the delay arm for the DLL configuration. Finally, Faraday rotating mirrors (FRMs) are used as the end mirrors of both the reference and delay arms to ensure polarization-independent interference.

At the Michelson interferometer output, each wavelength component (λ_1_ and λ_2_) is split by the FBG with 2 nm bandwidth and detected by photo-detectors. Each photo-detected power contains the frequency noise of the corresponding frequency modes (i.e., (*mf*_rep_ + *f*_ceo_ + 2 *f*_m_) for λ_1_ and (*nf*_rep_ + *f*_ceo_ + 2 *f*_m_) for λ_2_) weighted by the delay time (τ) in the form of phase noise at 2 *f*_*m*_ carrier frequency^38^. The obtained 100-MHz signals are filtered by RF band-pass filters with 24 MHz bandwidth, which produces >40 dB SNR in the 100-kHz resolution bandwidth. Finally, 100-MHz RF signals from each wavelength are amplified and frequency-mixed. The common frequency components (*f*_ceo_ + 2 *f*_m_) are rejected by a frequency mixer, and only the repetition-rate frequency noise, *δ(m* − *n) f*_rep_, is detected at the baseband. This signal can be applied to either the actuator in the comb (path A in Fig. 1, for PLL) or the PZT stretcher in the fibre delay line (path B in Fig. 1, for DLL).

To convert the measured voltage noise PSD at the mixer output to the equivalent frequency noise PSD, the following transfer function of the delay line should be divided.


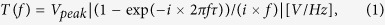


where *V*_*peak*_ is the amplitude of the low-pass filtered mixer output voltage from interference pattern, *f* is the Fourier frequency, and *τ* is the round-trip delay time between the two arms in the interferometer. When the Fourier frequency is far lower than 1/τ, the transfer function is simply a constant 2*πτV*_*peak*_ [V/Hz]. However, the detection sensitivity is nullified at Fourier frequencies of 1/τ and its harmonics. As a result, measurement noise artefact peaks appear in the jitter measurement results (as shown in Figs 2, 3, 4 and 5). As shown in Fig. 3, the trade-off between the background noise level and the null-point frequency position 1/τ can be achieved by selecting right fibre delay length. As the final step, we convert the frequency noise PSD at 2.5-THz (20-nm wavelength difference at 1550 nm) into the phase noise and timing jitter PSD, and further convert it to the phase noise at 10-GHz carrier for easy comparison with other state-of-the-art microwave source performances.

As a final note, in the measurement set-up design, total dispersion of the setup (including FBGs and fibre link) should be kept low enough to make sure the additional Gordon-Haus timing jitter^55^ does not hamper the measurement. In our experiment, the total dispersion was ~60 ps/nm (for 1-km fibre link case) and the additional Gordon-Haus timing jitter PSD for low-jitter mode-locked Er-fibre lasers was projected to be ~100 times lower than the measurement resolution floor. As a result it did not limit the measurement accuracy. If necessary, we can employ a DCF section in the fibre link to reduce the total dispersion.

### Mode-locked oscillator measurement experiment conditions

The home-built stretched-pulse Er-fibre oscillator has an optical spectrum centred at 1584 nm with 64-nm FWHM bandwidth. For the jitter measurement, 2-mW of optical power from the oscillator is used. It is filtered by FBGs centred at 1540 nm and 1560 nm for high-efficiency amplification with a C-band EDFA. The Origami-15 oscillator is tested with 3 mW optical power to the setup. We used 1550 nm and 1570 nm for Origami-15 because the optical spectrum is centred at 1562 nm with 15.6 nm FWHM bandwidth. The FC1500-250-ULN oscillator (with 57-nm 3-dB bandwidth centred at 1552 nm) is tested with 2 mW input optical power and FBGs centred at 1540 nm and 1560 nm.

### Supercontinuum measurement experiment conditions

The used supercontinuum source is a commercial Er-fibre-based frequency comb source (FC1500 from MenloSystem GmbH manufactured in 2010). It has a 250-MHz mode-locked oscillator at 1580-nm centre wavelength with 24-nm FWHM bandwidth. Supercontinuum is generated by the P250 unit and its spectrum spans over octave from 1 μm to 2 μm. For measuring the supercontinuum jitter, it is filtered by an optical band-pass filter centred at 1550-nm with 40-nm FWHM bandwidth, and the fibre-coupled average power is 850 μW. Two wavelengths at 1540 nm and 1560 nm (with 2-nm FWHM bandwidth) are used for both the oscillator and the supercontinuum jitter measurements.

## Additional Information

**How to cite this article**: Kwon, D. *et al*. Reference-free, high-resolution measurement method of timing jitter spectra of optical frequency combs. *Sci. Rep.* **7**, 40917; doi: 10.1038/srep40917 (2017).

**Publisher's note:** Springer Nature remains neutral with regard to jurisdictional claims in published maps and institutional affiliations.
